# Length-Dependent Modulation of B Cell Activating Factor Transcripts in Chicken Macrophage by Viral Double-Stranded RNA

**DOI:** 10.3390/vaccines11101561

**Published:** 2023-10-03

**Authors:** Magda I. Abo-Samaha, Mohammed M. Sharaf, Abeer F. El-Nahas, Solomon O. Odemuyiwa

**Affiliations:** 1 Department of Pathobiology, College of Veterinary Medicine, Tuskegee University, Tuskegee, AL 36830, USA; magda.abosamaha@alexu.edu.eg; 2 Department of Animal Husbandry and Animal Wealth Development, Faculty of Veterinary Medicine, Alexandria University, Alexandria 5410012, Egypt; sharafweb@yahoo.com (M.M.S.); abeer.elnahas@alexu.edu.eg (A.F.E.-N.); 3 Department of Veterinary Pathobiology, College of Veterinary Medicine, University of Missouri, Columbia, MO 65211, USA

**Keywords:** HD11, dsRNA, poly(I:C), macrophage, BAFF, chicken

## Abstract

Viral double-stranded RNA (dsRNA) interacts with Retinoic-acid-inducible-gene-1 (RIG-1)-like receptors (RLRs) to induce type 1 interferons. Melanoma-derived-antigen-5 (MDA-5), an RLR, but not RIG-1, is found in chickens. Ducks express both RIG-1 and MDA-5, a possible cause of differences in susceptibility to influenza virus infection between chickens and ducks. Using the HD11 chicken macrophage cell line and an RT^2^ Profiler PCR-array system, we showed that high-molecular-weight poly(I:C), HMW-poly(I:C), upregulates CCL4, interferon-gamma, interleukin-1, and interleukin-6 mRNA transcripts. HMW-poly(I:C), an in vitro surrogate of long dsRNA species, also induces the upregulation of IL-12B and B cell activating factor (BAFF). Conversely, low-molecular-weight poly(I:C), LMW-poly(I:C) did not induce a distinct cytokine expression pattern. Nonetheless, co-transfection of LMW and HMW-poly(I:C) significantly reduced the upregulation of IL12B and BAFF by HMW-poly(I:C). These findings support previous studies that found no expression of RIG-1, a receptor for short dsRNA species, in chicken cells. Surprisingly, however, our data suggested that in the absence of RIG-1 in chicken macrophages, short dsRNA species may inhibit macrophage-mediated B cell development and survival by modulating the expression of BAFF without significantly reducing type 1 interferon response.

## 1. Introduction

Viral infection leads to a cascade of early events triggered by activation of innate immunity that, among other functions, modifies the immediate tissue environment and dictates the texture and strength of subsequent adaptive immune response [1]. The tissue macrophage plays a central role in the orchestration of innate immunity and, following modification of the tissue environment, differentiates into classically (M1) or activated (M2) macrophages. Macrophages direct the immune response to viruses by producing cytokines and chemokines following the interaction of Pattern Recognition Receptors (PRR), expressed by macrophages, with pathogen-associated molecular patterns (PAMPs) expressed by viruses. The pro-inflammatory cytokine environment that ensues is crucial for priming the adaptive immune response [2]. Like mammalian macrophages, avian macrophages have been shown to alter their phenotype following specific cytokine stimuli and thereby play a significant role in the pathogenesis of several viral diseases in chickens. 

A major PAMP in RNA viruses is double-stranded RNA (dsRNA), an intermediate species during virus replication. Toll-like receptor 3 (TLR3) on the surface of macrophages serves as a PRR for extracellular dsRNA and those found in intracellular organelles [3]. Conversely, the main ligands for intracellular cytoplasmic dsRNA are RIG-like receptors (RLRs). The most widely studied RLRs are products of the Retinoblastoma Inducible Gene-1 (RIG-1) and Melanoma Differentiation-associated gene 5 (MDA-5) [4]. In general, short RNA species (less than 1.5 kbp in length) bind to the CARD domain on RIG-1 and induce a copious secretion of type I interferons. Conversely, MDA-5 interacts with long dsRNA species to induce the same effect [5,6]. 

Several studies indicated that while dsRNA is capable of inducing the type 1 interferon response in chicken cells, the degree of response is much lower than in cells derived from ducks [7,8]. This discrepancy was traced to the absence of RIG-1 in chicken cells, while duck cells express both RIG-1 and MDA-5. This crucial difference in the expression of pathogen recognition receptors was shown to affect the replication of the avian influenza virus in chicken cells. The relative resistance of ducks to disease induced by influenza virus when compared to the domestic chicken has been traced to the absence of RIG-1 in chicken cells [9]. 

In recent years, attempts to use poly(I:C), a synthetic analog of viral dsRNA, as an adjuvant in the design of viral vaccines for poultry produced variable results, suggesting that the MDA5 signaling pathway may function differently in species that do not express RIG-1 [10]. In the present study, we explored the activity of the MDA-5 signaling pathway in chickens by investigating the interaction of low-molecular-weight and high-molecular-weight poly(I:C) with chicken macrophages. The primary aim of the study is to determine how different species of dsRNA affect changes in the cytokine RNA transcripts induced in chicken macrophages. We hypothesized that the transcripts induced by long dsRNA species would be different from those induced by short dsRNA species. Our results showed that short, low-molecular-weight dsRNA species may modulate the ability of long dsRNA species to induce proinflammatory cytokines in avian macrophages. The data suggest that the impact of low- versus high-molecular-weight dsRNA may be important in cellular versus antibody response by modulating the transcription of the B cell activating factor gene in macrophages. These preliminary findings are important in understanding the pathogenesis of viral infections in chickens and may provide the basis for the design of new dsRNA-based vaccine adjuvants for inducing and boosting immune response in poultry.

## 2. Materials and Methods

### 2.1. Macrophages

The chicken macrophage cell line HD-11 was used in this study to avoid the inconsistencies initially associated with the heterogeneity among primary cultures of macrophages [11]. Previous studies showed that this cell line could reliably be used to model the functions of primary chicken macrophage cultures [12]. The cell line was a gift from Dr. Haiqi He of the Southern Plains Agricultural Research Center (USDA-ARS), College Station, TX, USA. The cells were cultured in RPMI-1640 supplemented with 10% heat-inactivated fetal bovine serum, 10 mM HEPES, 0.1 mM non-essential amino acids, 2 mM glutamine, 1 mM sodium pyruvate, 100 U/mL penicillin, and 100 µg/mL streptomycin (Sigma Aldrich Co., St. Louis, MO, USA). The cells were maintained at 42 °C in a 5% CO_2_ incubator.

### 2.2. Activation of Macrophages 

To study RIG-1/MDA-5 signaling in HD-11, a commercial transfection reagent that contains the TLR3/RLR ligand polyinosilic:polycytidilic acid (poly I:C) complexed to the commercial transfection reagent LyoVec (InvivoGen, San Diego, CA, USA) was used. Unlike naked poly(I:C), however, the poly(I:C) LyoVec reagent activates only the RIG-1/MDA-5 but not TLR3 pathways [13,14]. Both the high-molecular-weight poly(I:C) HMW/LyoVec and the low-molecular-weight Poly(I:C) LMW/Lyovec reagents were used in this study. Per the manufacturer’s instructions, the LyoVec reagents were reconstituted by adding 500 µL of warm medium to each vial to obtain a final concentration of 25 µg/mL poly(I:C) (LMW) and poly(I:C) (HMW), group 1 and group 2 stock poly(I:C) reagents, respectively. To generate a mixture containing the same concentration of poly(I:C) (LMW) and poly(I:C) (HMW) in the same vial, each vial was first reconstituted in 250 µL of medium and pooled into a single vial that constituted the group 3 poly(I:C) reagent. Cells were grown until confluent in a 24-well cell culture plate. After washing off detached cells with sterile phosphate-buffered saline (PBS), 2 mL of fresh culture medium was added. To activate the cells, 20 µL of stock poly(I:C) reagents (final concentration 250 ng/mL) were added to the wells to generate three different experimental treatment groups: Poly(I:C) LMW/LyoVec (Group 1), Poly(I:C) HMW/LyoVec (Group 2), and Poly(I:C) HMW + LMW/LyoVec (Group 3). The control group consisted of cells to which 20 µL of medium was added (LyovecControl). Cells were incubated overnight (10–12 h) at 42 °C. After carefully removing the medium, cells were washed with PBS and resuspended in lysis buffer (Qiagen, Valencia, CA, USA) before RNA extraction with on-column DNA digestion to remove chromosomal DNA (Qiagen RNeasy mini-kit). Total RNA was quantified using Nanodrop N-2000 (Agilent Biosystems, Santa Clara, CA, USA). Following assessment of RNA quality, first-strand cDNA synthesis was performed from 100 ng total RNA on an Eppendorf MasterCycler using a ReactionReady First Strand Synthesis kit (Qiagen, Germantown, MD, USA).

### 2.3. RT-PCR Array

The RT^2^ Profiler Array Chicken Innate and Adaptive Immune Responses system (Qiagen) was used to quantify changes in RNA transcripts in a 96-well format on a Stratagene Model Mx3005P unit (Agilent Technologies, Santa Clara, CA, USA). The RT^2^ Profiler PCR Array system is a fully validated pathway-focused panel of laboratory-verified qPCR assays with integrated, patented controls to enable quick, reliable gene expression analysis. The array contains a panel of 84 immune-related genes from the innate (Pattern Recognition Receptors, Cytokines, Other), adaptive (Th1, Th2, Th17, and Treg markers, T Cell Activation, Cytokines, Other), humoral, and inflammatory responses, as well as the defense response to bacteria and viruses. Nucleic acid extraction, reverse transcription, normalization with housekeeping genes, and data analysis were carried out according to fully validated protocols and Excel macros supplied by the manufacturers of the PCR Array System. Reproducibility was ensured by running samples in triplicates and pooling samples for RT-PCR array. This array system demonstrated strong correlations across technical replicates, lots, and instruments with average correlation coefficients >0.99, ensuring reliable detection of differences in expression between biological samples (Qiagen, Hilden, Germany).

## 3. Results and Discussion

**Figure 1:**
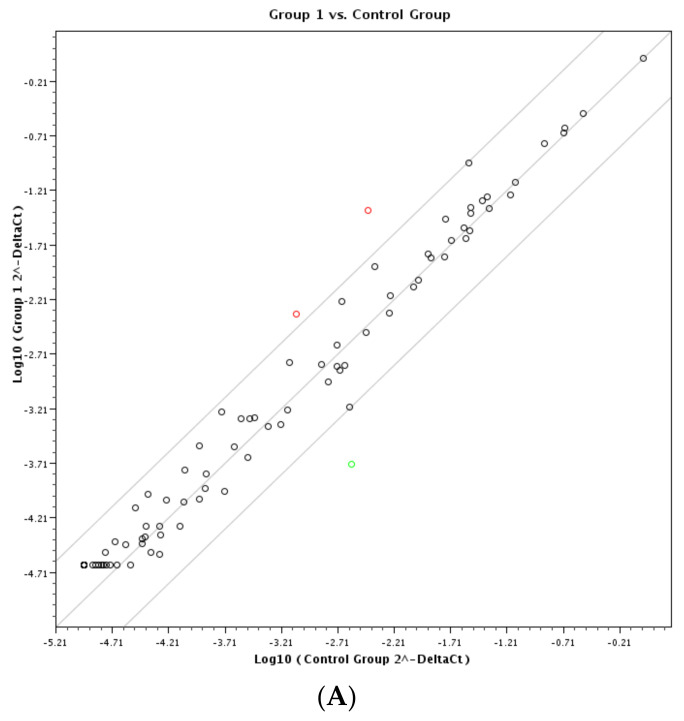

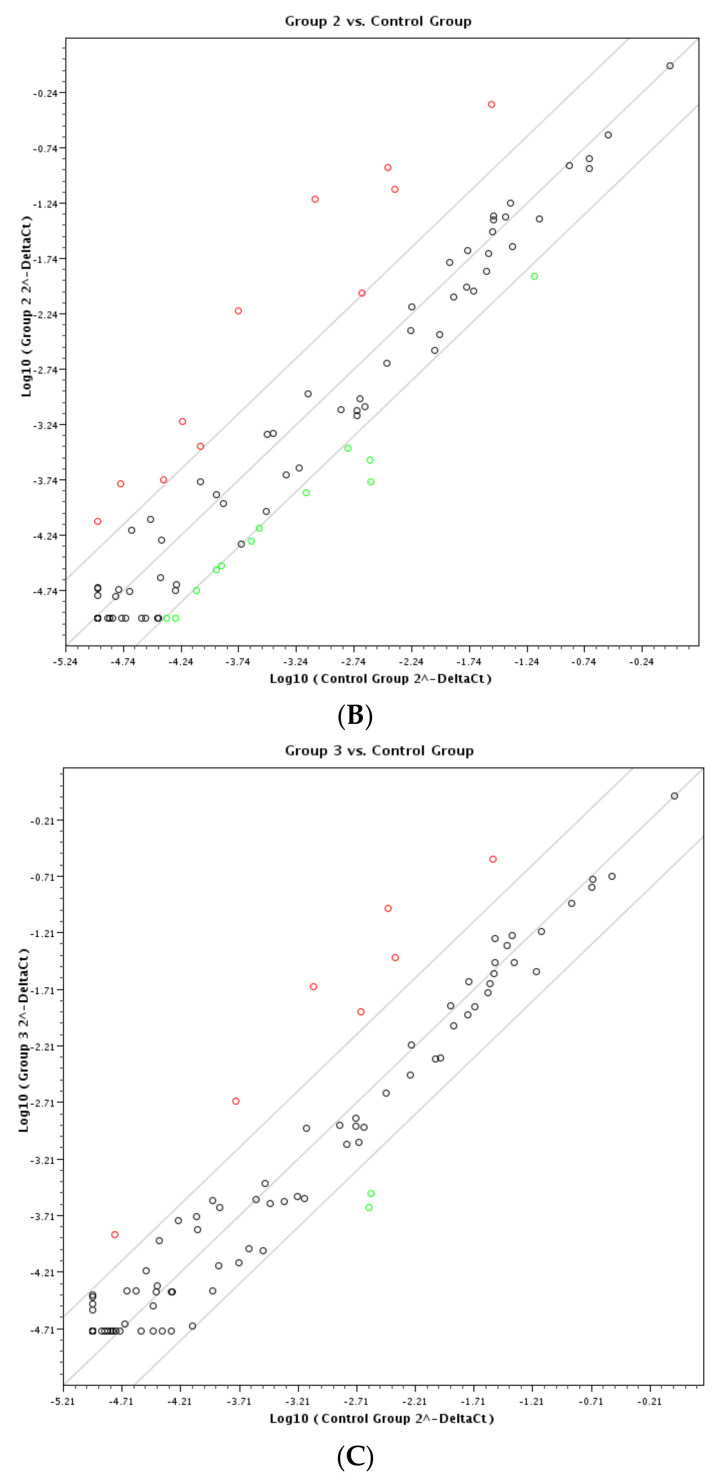
Changes in chicken macrophage gene expression following poly(I:C) treatment. Cultures of HD-11 chicken macrophages were transfected and incubated for 12 h with 250 ng/mL of control reagents, Control; LMW poly(I:C), group 1; HMW poly(I:C), group 2; or a mixture of LMW + HMW poly(I:C), group 3. Following extraction of mRNA from lysed cells, changes in the expression of 84 different cytokine and cytokine receptor genes were evaluated using the Qiagen RT2 RT-PCR gene array system. Changes in gene expression relative to control are presented for LMW poly(I:C) (**A**), HMW poly(I:C) (**B**), and LMW + HMW poly(I:C) (**C**). A minimum of a 4-fold change in gene expression is regarded as significant. In Figure (**A**–**C**), upregulated genes are indicated as open green circles. The further away the circle is from the outer boundary lines, the higher the fold change in the upregulated genes. Downregulated transcripts are shown as open red circles while upregulated genes are shown as open green circles.

The results of the RT-PCR array are summarized in Figure 1 and Table 1. 

An arbitrary minimum 4-fold change in gene expression, when each of groups 1, 2, and 3 were compared to the control group, was regarded as significant. Data analysis using manufacturer-provided Excel templates returned information on the reliability of gene expression data for each target. Only targets with high levels of reliability were selected. Compared to control samples, only two genes were upregulated (CCL4, +11x; CSF-3, +6x) following treatment of HD11 with LMW poly(I:C) (group 1). In contrast, treatment with HMW poly(I:C), which represents long viral dsRNA species, led to the upregulation of 11 different genes (group 2). Interestingly, compared to group 1, there is a three-fold higher upregulation of CCL4 expression and a 12-fold higher upregulation of CSF-3 expression in macrophages treated with high-molecular- versus low-molecular-weight poly(I:C). These results confirmed the previous finding of a differential effect of short and long dsRNA species on the activation of RLRs [5,6]. Since long dsRNA species were associated with activation of MDA-5, the marked effect of HMW-poly(I:C) on HD-11 cells supports the presence of MDA-5 but not RIG-1 in avian cells [7]. However, it is surprising that, like its high-molecular-weight counterpart, low-molecular-weight poly(I:C) still upregulated CCL4 and CSF-3 in our study. This would suggest that there is a signaling pathway activated by LMW poly(I:C) that is dependent on neither RIG-1 nor MDA-5 to induce the upregulation of CCL4 and CSF-3 transcripts. It has previously been suggested that there is a very wide diversity of RNA ligands for RIG-1; such promiscuity in the recognition of dsRNA species makes it possible that different folding patterns of even short dsRNA species might activate other innate signaling pathways in the absence of RIG-1 in chickens [15]. In addition, it has been shown that MDA-5 can initially bind short dsRNA species but rapidly discard them and prevent rebinding; this initial short-term binding may be enough to generate the signals that resulted in the gene expression changes observed in HD11 following LMW poly(I:C) treatment in our study [16]. 

It is currently unknown whether short dsRNA species could affect how long dsRNA species interact with MDA-5 in chickens. Starting with the hypothesis that in species that lack RIG-1, short dsRNA would influence the interaction of long dsRNA with innate signaling, we transfected a mixture of LMW-poly(I:C) and HMW-poly(I:C) into HD11 cells. Our results showed that in this group (group 3), the number of genes upregulated by HMW-poly(I:C) was reduced from 11 to 7; no changes in the expression of IL-6, IL-12B, CSF-2 (GM-CSF), and TNSF13B (B cell Activating Factor; BAFF) were observed. Most importantly, our data showed that BAFF was upregulated in the HD11 macrophage cell line following treatment with HMW poly(I:C) alone but not when transfected along with LMW poly(I:C). The TNF family cytokine TNSF13B, otherwise called B cell activating factor (BAFF), is an important B cell survival factor that is crucial in the generation of antibody response in chickens [17,18]. The upregulation of BAFF supports the idea that the early adjuvant effect of HMW poly(I:C) is important for priming both T cell- and B cell-dependent adaptive immune response. Similarly, the importance of IL12B in antibody-mediated immune response in chickens is underscored by studies that showed a strong association between variations in the generation of antibodies across different breeds of chicken with single nucleotide polymorphisms in the chicken IL12B gene [19,20]. In a mouse model of B cell-mediated lupus-like autoimmunity, poly(I:C) was found to inhibit B cell function by inducing Pellino1(Peli1), a negative regulator of non-canonical NF-kB activation and antibody synthesis [21]. Although comparable to our current result suggesting that poly(I:C) may modulate the upregulation of BAFF transcripts and possibly inhibit B cell-mediated immune response, mice possess both RIG-1 and MDA-5. The direct effect of poly(I:C) on B cells from chickens, a species that expresses only MDA-5, is currently unknown. Nonetheless, because of the induction of type I interferons and proinflammatory cytokines by poly(I:C), it is used as a clinically approved vaccine adjuvant for cancer immunotherapy and antiviral immunity [22,23]. This use of poly(I:C) is largely based on its interaction with TLR3 [24]. It is conceivable that inhibition of BAFF transcripts by abundant short low molecular weight dsRNA could be one of the mechanisms through which viruses escape antibody-mediated immune response [24]. 

Based on our preliminary data, we conclude that short dsRNA species, represented by LMW-poly(I:C), induce a different profile of cytokine RNA transcripts than long dsRNA species represented by HMW-poly(I:C). Using a mixture of both species, we have provided data that suggest that short dsRNA species may affect the biological function of long dsRNA species. The profile of cytokine transcripts in this mixture suggests that short dsRNA species may influence the long dsRNA priming of the antibody-mediated immune response by affecting the transcription of the B cell activating factor gene. Ongoing studies are exploring how changes in the lengths of viral RNA species could affect cytokine transcripts in the primary culture of avian macrophages and regulate antibody production in chickens infected with RNA viruses. 

## Figures and Tables

**Table 1:** Effects of poly(I:C) stimulation on cytokine RNA transcripts in chicken macrophage cell line HD11. Numbers represent fold upregulation relative to sham-treated control cells.

Cytokine Gene	Fold Increase in mRNA Expression Following Treatment		
	LMW Poly(I:C)	HMW Poly(I:C)	LMW + HMW Poly(I:C)
CCL1	<4	34	11
CCL4	11	34	28
CCL5	<4	4	6
CSF3	6	74	25
IFNG	<4	10	8
IL1B	<4	19	9
IL8	<4	16	10
IL6	<4	7	<4
IL12B	<4	4	<4
CSF2	<4	5	<4
TNSF13B (BAFF)	<4	10	<4

## Data Availability

Not applicable.
